# The adipokine lipocalin-2 in the context of the osteoarthritic osteochondral junction

**DOI:** 10.1038/srep29243

**Published:** 2016-07-07

**Authors:** Amanda Villalvilla, Adela García-Martín, Raquel Largo, Oreste Gualillo, Gabriel Herrero-Beaumont, Rodolfo Gómez

**Affiliations:** 1 Bone and Joint Research Unit, IIS-Fundación Jiménez Díaz, UAM, Avda Reyes Católicos, Madrid, 28040, Spain; 2 Department of Bioengineering, Universidad Carlos III de Madrid, CIEMAT-CIBERER, IIS-Fundación Jiménez Díaz, Madrid, 28040, Spain; 3 Research Laboratory 9 (NEIRID LAB), Institute of Medical Research, SERGAS, Santiago University Clinical Hospital, Santiago de Compostela, 15706, Spain; 4 Musculoskeletal Pathology Laboratory, Institute IDIS, Santiago University Clinical Hospital, Santiago de Compostela, 15706, Spain

## Abstract

Obesity and osteoarthritis (OA) form a vicious circle in which obesity contributes to cartilage destruction in OA, and OA-associated sedentary behaviour promotes weight gain. Lipocalin-2 (LCN2), a novel adipokine with catabolic activities in OA joints, contributes to the obesity and OA pathologies and is associated with other OA risk factors. LCN2 is highly induced in osteoblasts in the absence of mechanical loading, but its role in osteoblast metabolism is unclear. Therefore, because osteochondral junctions play a major role in OA development, we investigated the expression and role of LCN2 in osteoblasts and chondrocytes in the OA osteochondral junction environment. Our results showed that LCN2 expression in human osteoblasts and chondrocytes decreased throughout osteoblast differentiation and was induced by catabolic and inflammatory factors; however, TGF-β1 and IGF-1 reversed this induction. LCN2 reduced osteoblast viability in the presence of iron and enhanced the activity of MMP-9 released by osteoblasts. Moreover, pre-stimulated human osteoblasts induced LCN2 expression in human chondrocytes, but the inverse was not observed. Thus, LCN2 is an important catabolic adipokine in osteoblast and chondrocyte metabolism that is regulated by differentiation, inflammation and catabolic and anabolic stimuli, and LCN2 expression in chondrocytes is regulated in a paracrine manner after osteoblast stimulation.

Obesity and osteoarthritis (OA) show a reciprocal relationship. Among the comorbidities associated with obesity, OA is of special interest because this disease is a contributing factor to weight gain^1^,^2^. OA is the most common rheumatic disease and is characterized by progressive degradation of the articular cartilage^3^ and by severe alterations such as loss of joint architecture, pain, and disability, which significantly contribute to sedentary behaviour^1^. This sedentary lifestyle is a well-known factor associated with weight gain and obesity^2^, which enhances cartilage degradation due to mechanical joint overload^4^ and altered metabolism^4^, including dysregulated adipokine production^5^.

Although OA is considered to be a disease of the whole joint, there is a growing interest in the OA alterations that affect the functional unit formed by the articular cartilage and the underlying subchondral bone^6^. Indeed, both tissues work together to dissipate the mechanical stress that results from joint movement. However, subchondral bone also contributes to the maintenance of cartilage homeostasis and integrity^6^,^7^. Accordingly, biochemical and structural alterations in the subchondral bone may alter cartilage function and load-bearing distribution, which in turn may promote cartilage degradation^6^,^8^,^9^. Likewise, abnormal mechanical loading and biochemical alterations in the cartilage are associated with aberrant metabolism in the subchondral bone, including the production of pro-inflammatory factors and the overexpression of certain anabolic factors^8^,^10^.

OA joint inflammation is mediated, in part, by innate immune responses elicited by molecules such as interleukin-1 beta (IL-1β) and Toll-like receptor 4 (TLR4) agonists (host-derived molecules generated upon tissue damage)^11^. The activities of these molecules affect both the articular cartilage and the subchondral bone^11^,^12^ and are associated with cartilage degradation^11^,^13^, changes in osteoblast phenotype^11^,^14^, and alterations in the bone-cartilage crosstalk^11^,^14^. In light of these actions, others have suggested that inflammatory and catabolic factors present in the OA cartilage can reach the OA subchondral bone, and the ones present in the OA subchondral can also reach the OA cartilage^12^,^14^,^15^. Although the crosstalk between these tissues has been investigated, a limited number of *in vitro* mechanistic studies have been performed, and thus the precise factors involved in the molecular crosstalk between chondrocytes and osteoblasts are poorly understood.

At the crossroads of several OA risk factors, such as obesity, altered mechanical loading, joint inflammation and ageing, is lipocalin-2 (LCN2)^5^,^16^, a mechanoresponsive adipokine induced by pro-inflammatory factors in joint tissues, whose circulating levels are elevated in obese and aged individuals^5^,^17^,^18^,^19^. LCN2 is a 25 kDa glycoprotein that forms covalent complexes with matrix metalloproteinase 9 (MMP-9)^20^. LCN2 expression has been investigated in joint tissues^21^,^22^,^23^,^24^. In chondrocytes, LCN2 expression is induced upon stimulation with inflammatory factors^21^. Accordingly, increased levels of LCN2 have been found in OA synovial fluid (SF)^22^,^23^,^25^ and OA cartilage^25^. Consistently with this observation, LCN2 promotes cartilage breakdown by blocking MMP-9 auto-degradation^22^ and by reducing chondrocyte viability^23^,^26^,^27^. In contrast to its role in cartilage, information about the role of LCN2 in bone metabolism is limited. LCN2 expression in osteoblasts is increased by tumour necrosis factor (TNF) and IL-17^28^ and in the absence of mechanical force stimulation^24^. Additionally, elevated circulating levels of LCN2 have been correlated with an increased risk of osteoporotic fractures in aged individuals^18^, and mice overexpressing LCN2 are smaller than their wild-type littermates and exhibit bone alterations^29^.

The subchondral bone plays a major role in OA pathophysiology. Therefore, considering the catabolic activities of LCN2 in other joint tissues, we investigated the role of LCN2 in osteoblast metabolism by mimicking the OA osteochondral junction environment. Our results revealed that LCN2 expression was decreased throughout osteoblast differentiation but was induced by catabolic and inflammatory factors. Conversely, anabolic factors blocked this induced expression. LCN2 affected osteoblast viability and promoted MMP-9 activity. Furthermore, stimulated osteoblast conditioned medium induced LCN2 expression in human chondrocytes, whereas LCN2 induction in these cells was inhibited by anabolic factors.

## Materials and Methods

### Reagents

Foetal bovine serum (FBS), alpha-MEM medium, Dulbecco’s modified Eagle’s medium (DMEM), antibiotics, trypsin, and L-glutamine were purchased from Lonza (Verviers, Belgium). IL-1β and IGF-1 were purchased from PeproTech (Rocky Hill, NJ, USA). Dexamethasone (Dx) was obtained from Merck & Co. (Kenilworth, NJ, USA). Collagenase IV, lipopolysaccharide (LPS; *E. coli* 055:B5), L-ascorbic acid, β-glycerophosphate, transforming growth factor-beta 1 (TGF-β1), methyl-thiazolyl-tetrazolium (MTT) dye, and ferric ammonium citrate were purchased from Sigma-Aldrich (St. Louis, MO, USA). Recombinant mouse LCN2 and goat anti-human LCN2 antibodies were obtained from R&D Systems (Minneapolis, MN, USA). Unless otherwise specified, all reagents were purchased from Sigma-Aldrich.

### Cell culture

Human tissues were obtained from patients who had undergone total knee replacement surgery. The Ethics Committee for Clinical Research at the Fundación Jiménez Díaz (FJD) Institute approved the protocol, and written informed consent was obtained from all patients. All processes were carried out according to relevant guidelines and regulations. Human chondrocytes were obtained as previously described^30^. Human osteoblasts were obtained by culturing bone pieces in culture dishes with DMEM containing 20% heat-inactivated FBS, 2 mM glutamine, and 100 U/ml of penicillin/streptomycin, and experiments were performed using cells at the third passage.

Mouse osteoblast precursor MC3T3-E1 cells were grown in alpha-MEM with 10% inactivated FBS, 4 mM glutamine, and 100 U/ml of penicillin/streptomycin. To induce differentiation, cells were seeded at 2.5 × 10^4^ cells/cm^2^, and the growth medium was exchanged with differentiation medium containing normal medium supplemented with 50 μg/ml L-ascorbic acid and 10 mM β-glycerophosphate.

### Cell treatment

Human primary cells were seeded onto 6-well plates and grown until confluence. MC3T3 cells (2 × 10^5^ cells per well) were also seeded onto 6-well plates. After culturing under serum-free conditions, or under 1% heat-inactivated FBS for human osteoblasts, cells were treated for 48 h with conditioned medium (CM) or the following stimuli alone or in combination: 1 μg/ml LPS, 1 ng/ml IL1β, 10 μM Dx, 10 ng/ml TGF-β1 (selected dose to mimic the overproduction observed in OA subchondral bone and to promote signalling through SMADs 2/3 and 1/5/8^31^), and 100 ng/ml IGF1. MC3T3 cells were also differentiated for 15 days and then treated with 10 μM Dx for 48 h as a de-differentiation factor for osteoblasts.

To obtain CM, cells were stimulated with IL-1β for 24 h and washed three times with serum-free medium. Fresh serum-free medium was subsequently added, and cells were cultured for 24 h. Then, CM was collected, sterile-filtered to remove cell debris and frozen until use.

### Cell transfection

MC3T3 cells were transfected with pCMV6-AC-GFP or pCMV6-LCN2-GFP (OriGene Technologies Inc., Rockville, MD, USA) using the X-tremeGENE HP DNA Transfection Reagent (Roche Applied Science, Mannheim, Germany) according to the manufacturer’s instructions. Briefly, 2 × 10^5^ cells/well were seeded onto 6-well plates and grown for 24 h. Then, the culture medium was replaced with serum-free medium, and cells were transfected using 3 μl of reagent and 1 μg of DNA per well. After 24 h of incubation, the transfection medium was replaced with growth medium. Then, the cells were cultured for 24 h, and CM was collected, sterile-filtered, and frozen. The transfected cells were lysed with Tripure reagent (Roche Diagnostics, Indianapolis, IN, USA) for RNA isolation.

### Gene expression analysis

After treatment, cells were lysed in Tripure reagent or RP1 buffer provided with a Nucleospin RNA/Protein Isolation Kit (Macherey-Nagel, Düren, Germany), and RNA was isolated according to the manufacturer’s protocol. cDNA was synthesized using a High-Capacity cDNA Reverse Transcription Kit (Applied Biosystems, Life Technologies, Grand Island, NY, USA). The mRNA expression levels of lipocalin-2 (LCN2), alkaline phosphatase (ALP), runt-related transcription factor 2 (Runx2), and bone gamma-carboxyglutamate protein (osteocalcin) were determined by real-time PCR using TaqMan gene expression assays (Applied Biosystems). The data were normalized using hypoxanthine phosphoribosyltransferase (HPRT) for human cells and glyceraldehyde 3-phosphate dehydrogenase (GAPDH) for murine cells, and data were expressed as the fold-change versus the unstimulated control or as a percentage of stimulated cells.

### Western blot analysis

Proteins were isolated using a Nucleospin RNA/Protein Isolation Kit. Twenty-five micrograms of protein from each sample was loaded and resolved on a 10% SDS-PAGE gel. Proteins were subsequently transferred to a polyvinyl difluoride (PVDF) membrane. To avoid interference between antibodies, the membranes were cut according to the molecular weight of the studied proteins and incubated with the corresponding antibody: goat anti-human LCN2 antibody (the lower part) or anti-human actin (the upper part). The antibody binding was visualized by enhanced chemiluminescence with the corresponding peroxidase-conjugated secondary antibody and Immobilon Western Detection kit (EMD Millipore, Billerica, MA, USA). To confirm equal loading of each sample, the expression of LCN2 was normalized to actin expression. The images were captured using ImageQuant LAS 4000 (GE Healthcare Life Sciences, Piscataway, NJ, USA) and analysed with ImageJ software (ImageJ v1.45s, NIH, Bethesda, MD, USA).

### Cell viability assay

Viability was tested using the MTT reagent. To perform the assay, 3 × 10^3^ cells/well were plated onto 96-well plates and treated with CM from cells that were non-transfected or transfected with either LCN2 or vector plasmid MC3T3, in the presence or absence of 0.5 μg/ml or 1 μg/ml of ferric ammonium citrate (FCA) for 68 h. Then, 0.5 mg/ml of MTT reagent was added, and after 4 h of incubation, formazan salt was dissolved in dimethyl sulfoxide (DMSO). Absorbance was measured at 570 nm with an Infinite 200 microplate reader (Tecan Group Ltd., Männedorf, Switzerland).

### Zymography

LCN2 stabilization of MMP-9 activity was determined by gelatine zymography. CM from MC3T3 cells was collected to detect MMP-9 activity. CM was mixed with increasing concentrations of recombinant mouse LCN2 and incubated for 1 h at 37 °C. Then, equal volumes were loaded onto a 10% polyacrylamide gel containing 0.1% gelatine, and proteins were separated by SDS/PAGE under non-reducing conditions at 4 °C. After this electrophoresis, the gels were washed with 2.5% Triton X-100 and incubated with proteolysis buffer (50 mM Tris–HCl pH 7.6, 0.2 mM NaCl, and 5 mM CaCl_2_, 0.2% NP-40 and 0.01% Tween 20) for 20 h at 37 °C. Next, the gels were incubated with staining buffer (0.05% Coomassie blue, 50% methanol, and 10% acetic acid), followed by destaining buffer (4% methanol and 8% acetic acid). The gels were scanned using a Gel Doc EZ scanner (Bio-Rad, Hercules, CA, USA) and analysed and quantified using ImageJ (ImageJ v1.45s).

### Statistical analysis

Data are expressed as the mean ± standard error of the mean (SEM) for at least three independent experiments. Significant differences were assessed using a one-way analysis of variance (ANOVA) or the Kruskal-Wallis test, followed by a Bonferroni or Dunn post-test, respectively, or Student’s t test or Mann-Whitney test when appropriate. All analyses were performed using Prism software (GraphPad Software Inc., La Jolla, CA, USA), and p < 0.05 was considered significant.

## Results

### LCN2 expression in osteoblast differentiation

**Figure 1:**
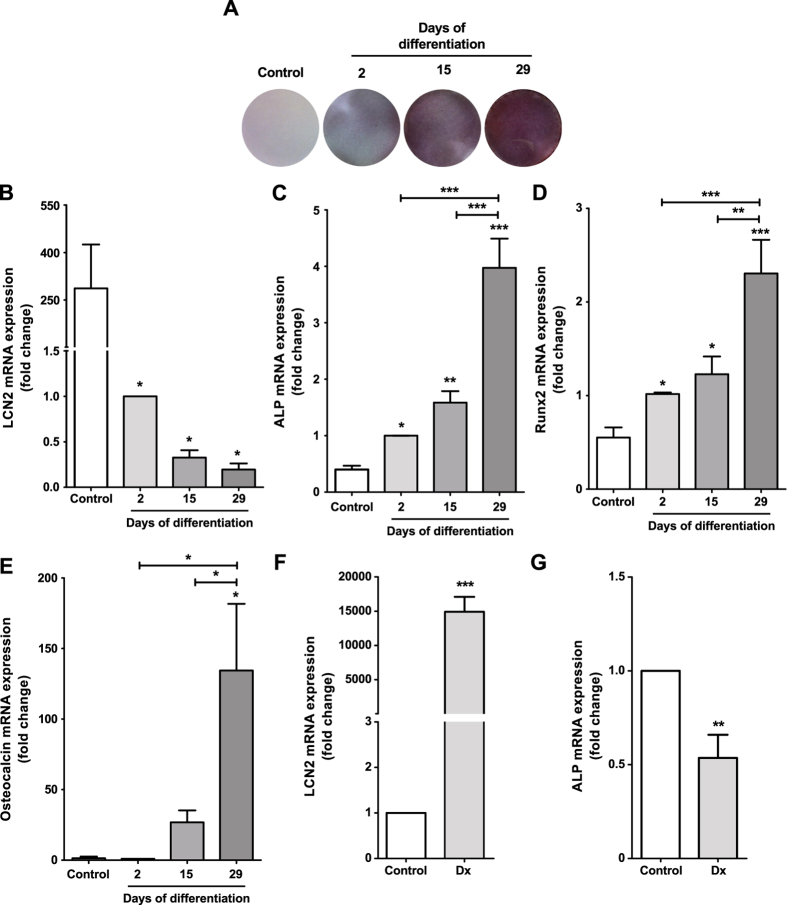
Evaluation of MC3T3 cell differentiation. MC3T3 cells were differentiated for 2, 15 or 29 days in the presence of 50 μg/ml L-ascorbic acid and 10 mM β-glycerophosphate. Then, matrix mineralization was evaluated using alizarin red staining (**A**), and LCN2 (**B**), ALP (**C**), Runx2 (**D**) and osteocalcin (**E**) gene expression was evaluated by real-time PCR. MC3T3 cells were differentiated for 15 days as described above and then were treated for 48 h with the de-differentiating factor 10 μM dexamethasone (Dx) to measure LCN2 (**F**) and ALP (**G**) gene expression. The results are presented as the mean ± SEM of at least three independent experiments. *p < 0.05; **p < 0.01; ***p < 0.001.

LCN2 is involved in the differentiation of several cell types^21^,^32^. Because osteoblasts from OA subchondral bone exhibit altered differentiation^12^, we investigated the expression of LCN2 during osteoblast differentiation. LCN2 expression was inhibited throughout the differentiation of the mouse osteoblastic MC3T3 cells, and was negatively related to matrix mineralization and to the expression of osteoblastic markers (Fig. 1). However, neither exogenous LCN2 addition nor transient LCN2 overexpression in these cells affected the expression of osteoblastic markers (data not shown). Hence, we tested whether LCN2 expression was associated with osteoblast de-differentiation by studying the effect of a known inhibitor of osteoblast differentiation^33^, specifically a high dose of Dx (10 μM), on LCN2 expression. MC3T3 cells differentiated for 15 days showed induced LCN2 expression (Fig. 1F) and inhibited ALP expression (Fig. 1G) after Dx treatment.

### LCN2 expression is enhanced by inflammatory/catabolic factors

**Figure 2:**
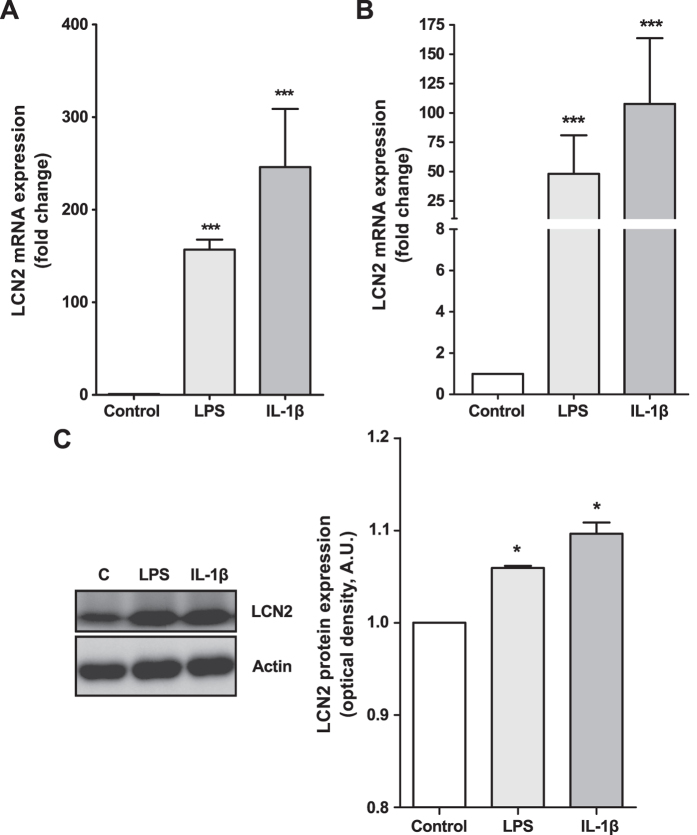
Effect of pro-inflammatory stimuli on LCN2 expression in murine and human osteoblasts. Non-differentiated MC3T3 cells were stimulated with 1 μg/ml LPS or 1 ng/ml IL-1β for 48 h to evaluate LCN2 gene expression (**A**). Human osteoblasts were grown until confluence and, after overnight culture in 1% FBS, cells were stimulated with 1 μg/ml LPS or 1 ng/ml IL-1β for 48 h. LCN2 mRNA expression was measured by real-time PCR (**B**). LCN2 protein expression was determined by western blot analysis and quantified by densitometry. Cropped images of the blots are shown. Full-length blots are presented in Supplementary Fig. 1. Gels were run under the same experimental conditions. (**C**). The results are presented as the mean ± SEM of at least four independent experiments and expressed as the fold-change over the control (*p < 0.05, ***p < 0.001).

LCN2 is a catabolic adipokine induced by inflammatory stimuli in several joint tissues^27^. Therefore, given that OA subchondral bone is exposed to multiple inflammatory and catabolic factors, we stimulated MC3T3 cells for 48 h with joint inflammatory factors, IL-1β (1 ng/ml) or the TLR4 agonist LPS (1 μg/ml), to test their effect on LCN2 expression. Both factors strongly enhanced the expression of LCN2 in these cells (Fig. 2A). Consistently with these results, IL-1β and LPS also induced the expression of LCN2 mRNA and protein in human primary osteoblasts (Fig. 2B,C). Given the anti-inflammatory properties of Dx and its catabolic activities at high concentration, we studied the combined effect of these inflammatory factors and Dx (10 μM) on LCN2 expression. Independently of the anti-inflammatory properties of Dx^21^, IL-1β and LPS synergized with Dx in the induction of LCN2 expression in MC3T3 cells (Supplementary Fig. S2) and in human primary osteoblasts (Supplementary Fig. S3).

### Effects of LCN2 on osteoblast viability and MMP-9 activity

**Figure 3:**
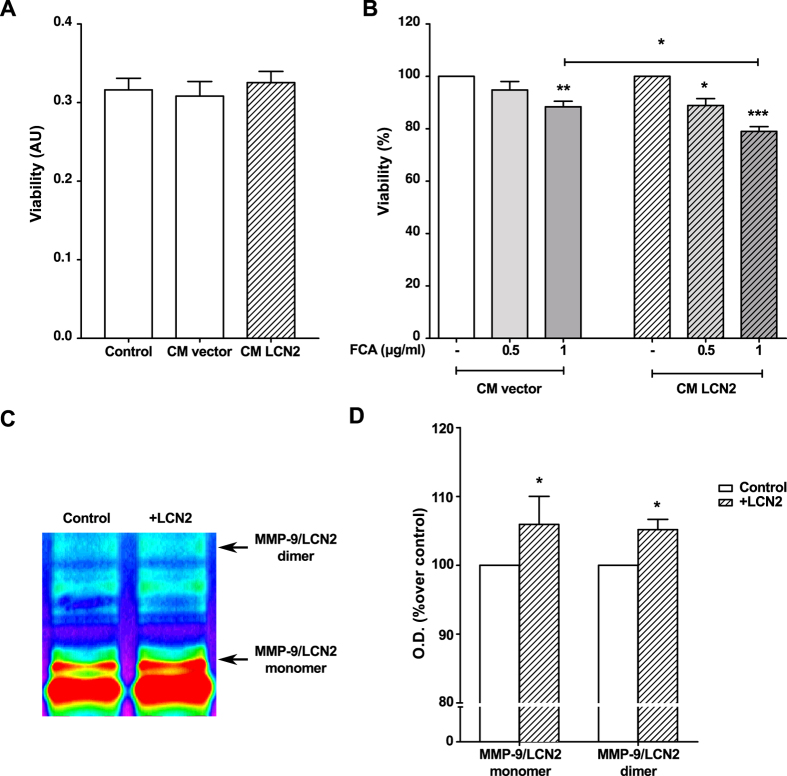
Effects of LCN2 gene expression in MC3T3 cells. Twenty-four hour CM from MC3T3 cells transfected with empty vector (CM vector) or LCN2-containing plasmid (CM LCN2) was used to stimulate MC3T3 cells for 48 h. The effect of LCN2-enriched medium on cell vitality was determined by the MTT assay in the absence (**A**) or presence (**B**) of an iron donor (ferric citrate ammonium, FCA) at 0.5 or 1 μg/ml. Additionally, 30 ng/μl of recombinant mouse LCN2 was incubated for 1 h at 37 °C with MC3T3 cell CM to detect the effect of LCN2 on MMP-9 activity by gelatine zymography. A cropped image of the gel is shown. The full-length gel is presented in Supplementary Fig. 6. (**C**). Gelatinolytic activity was measured by densitometry for both the MMP-9/LCN2 monomer and dimer (**D**). The results are presented as the mean ± SEM of at least three independent experiments and expressed as a percentage of the non-stimulated control (**B,D**) (*p < 0.05, **p < 0.01, ***p < 0.001).

LCN2 has been associated with cell viability regulation^23^,^26^,^27^. Therefore, to evaluate the potential consequences of the increased expression of LCN2 mediated by inflammatory stimuli, we investigated the effects of LCN2 on osteoblast viability. To mimic the induction of LCN2 observed in the stimulated osteoblasts and to isolate its effects on their viability, we generated LCN2-enriched medium by overexpressing LCN2 in MC3T3 cells (Supplementary Fig. S4). This overexpression did not affect the cell viability (data not shown). Likewise, the culture of MC3T3 osteoblasts in the obtained LCN2-enriched medium also did not affect cell viability (Fig. 3A). However, given the role of LCN2 on iron metabolism^34^ and the role that iron plays in osteoblast viability^35^,^36^, we found that LCN2-enriched medium significantly enhanced the inhibition of the osteoblast viability caused by the iron donor FCA (Fig. 3B).

LCN2 protects MMP-9 against auto-degradation, thus enhancing its catabolic activities^22^. Because MMP-9 is involved in bone remodelling, including in the OA subchondral bone^37^,^38^, we used gelatine zymography to determine whether exogenous LCN2 would enhance the MMP-9 activity present in the CM from MC3T3 cells. Incubation of this CM with a suitable dose of LCN2 (30 ng/μl), selected after dose-response experiments (Supplementary Fig. S5), induced an increase in MMP-9 activity at the band sizes corresponding to the monomeric and dimeric forms of the complex LCN2/MMP-9 (Fig. 3C,D).

### LCN2 expression is inhibited by anabolic factors in osteoblasts

**Figure 4:**
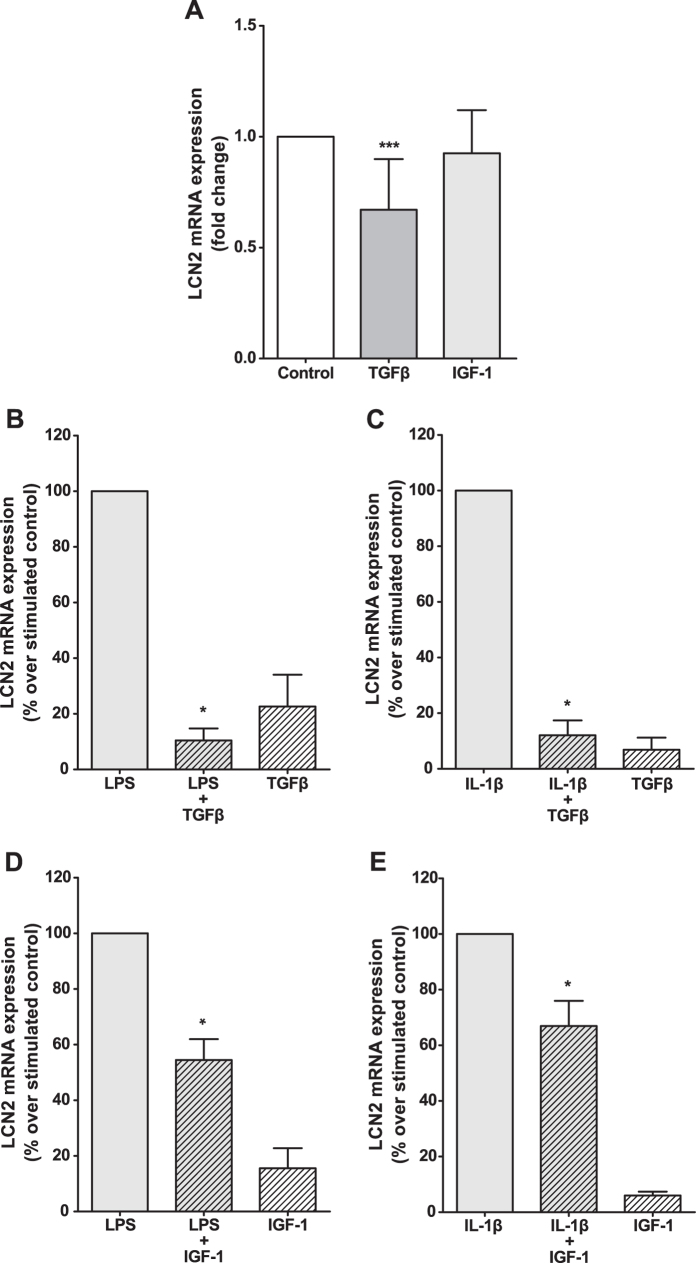
Effect of anabolic factors on LCN2 expression in primary human osteoblasts. Human osteoblasts were grown until confluence. After overnight culture in 1% FBS, cells were stimulated with 10 ng/ml TGF-β1 or 100 ng/ml IGF-1 alone (**A**) or in combination with 1 μg/ml LPS (**B,D**) or 1 ng/ml IL-1β (**C,D**) for 48 h. Then, LCN2 mRNA expression was measured by real-time PCR. The results are presented as the mean ± SEM of at least four independent experiments and expressed as a fold-change over the control (**A**) or as the percentage over the stimulated control (**B–E**) (*p < 0.05, ***p < 0.001).

Mechanical loading enhances osteoblast survival and bone formation^24^,^39^. Because LCN2 expression is highly induced in the absence of this stimulus^24^, we investigated whether IGF-1 and TGF-β1, two bone anabolic factors often related to OA pathophysiology and bone mechanical loading^8^,^40^,^41^,^42^, would modulate LCN2 expression in human osteoblasts. Unlike TGF-β1, IGF-1 did not inhibit LCN2 basal expression (Fig. 4A). However, TGF-β1 and IGF-1 stimulation of osteoblasts inhibited IL-1β- and LPS-mediated LCN2 expression (Fig. 4B–E).

### Effect of osteoblast-chondrocyte bidirectional crosstalk on LCN2 expression

**Figure 5:**
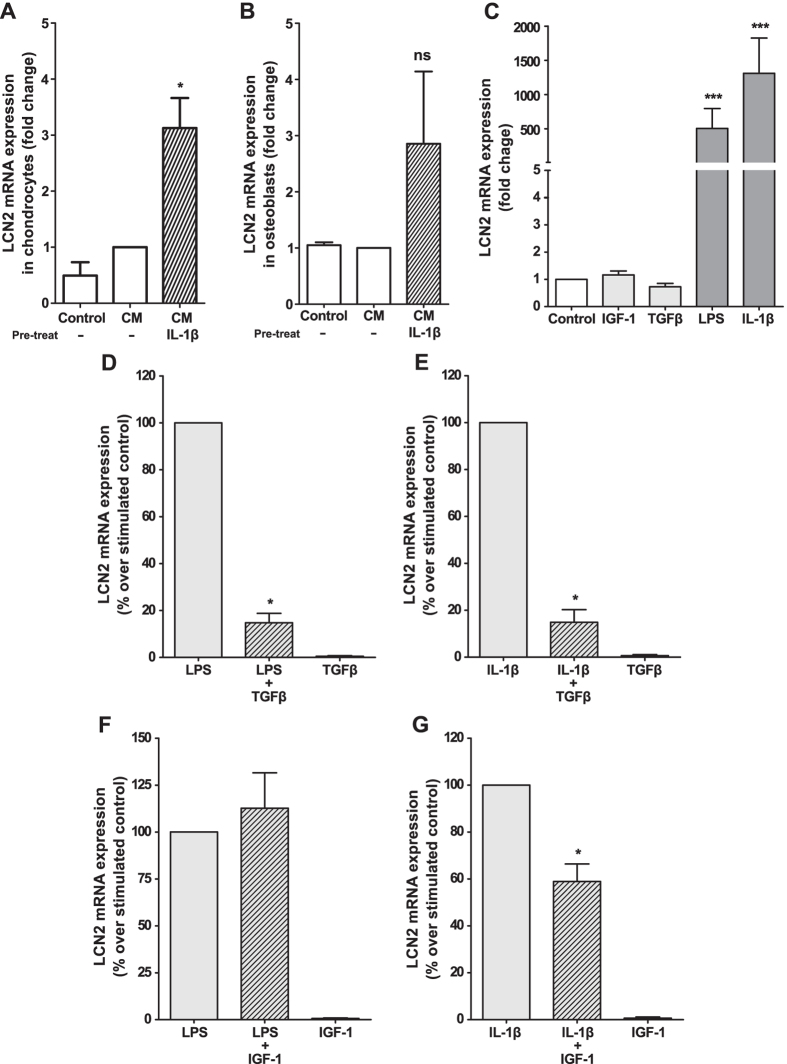
Modulation of LCN2 in osteoblast-chondrocyte crosstalk and in primary human chondrocytes. Primary human osteoblasts and chondrocytes were grown until confluence and stimulated with 1 ng/ml IL-1β for 24 h. After washing, CM was collected and used to stimulate human chondrocytes (**A**) or osteoblasts (**B**). Primary human chondrocytes were grown until confluence and then stimulated with 10 ng/ml TGF-β1, 100 ng/ml IGF-1, 1 μg/ml LPS or 1 ng/ml IL-1β alone (**C**) or with combined anabolic and pro-inflammatory factors (**D–G**) for 48 h. LCN2 mRNA expression was measured by real-time PCR. The results are presented as the mean ± SEM of at least four independent experiments and expressed as the fold-change over the non-stimulated CM (**A,B**), non-stimulated control (**C**) or as the percentage over the stimulated control (**D–G**). (*p < 0.05, ***p < 0.001, ns: non-significant).

The subchondral bone and the articular cartilage are tightly related. Therefore, we studied whether the crosstalk between osteoblasts and chondrocytes would affect the expression of LCN2. Accordingly, we treated human chondrocytes with CM from osteoblasts pre-stimulated with IL-1β or treated human osteoblasts with the CM from chondrocytes pre-stimulated with IL-1β. The CM from the pre-stimulated osteoblasts induced the expression of LCN2 in chondrocytes (Fig. 5A). In contrast, the CM from the pre-stimulated chondrocytes did not induce any significant changes in the expression of LCN2 in osteoblasts (Fig. 5B).

### LCN2 expression is modulated by inflammatory and anabolic factors in chondrocytes

OA cartilage is usually exposed to a pro-inflammatory environment that may enhance LCN2 expression and, therefore, cartilage degradation^22^,^27^. Consistently with this assumption, IL-1β and LPS stimulation induced the expression of LCN2 in human primary chondrocytes (Fig. 5C). Moreover, given the key role of several growth factors in OA pathophysiology, we investigated whether LCN2 expression in these cells was also modulated by IGF-1 and TGF-β1. Interestingly, none of these growth factors inhibited the basal expression of LCN2 in chondrocytes (Fig. 5C). Nonetheless, TGF-β1 inhibited the expression of LCN2 induced by IL-1β or LPS (Fig. 5D,E), whereas IGF-1 inhibited only IL-1β-mediated LCN2 expression (Fig. 5F,G).

## Discussion

In this work, we present evidence indicating that the expression of the obesity-related adipokine LCN2 is inhibited throughout osteoblast differentiation and is induced in osteoblasts and chondrocytes exposed to OA-related inflammatory stimuli. However, this induction was strongly blocked by IGF-1 and TGF-β1. We further show that LCN2 decreases the viability of iron-overloaded osteoblasts and enhances the activity of the osteoblast-released MMP-9. Finally, we show that CM from osteoblasts stimulated with an inflammatory factor (IL-1β) enhances the expression of LCN2 in chondrocytes.

Obesity and OA are two closely related pathologies that form a vicious cycle. Obesity promotes cartilage degradation due to excessive mechanical loading and an aberrant metabolic environment^4^,^5^, whereas cartilage degradation involves joint failure, pain and disability, which are associated with a sedentary lifestyle^1^. Indeed, sedentary behaviour is linked to weight gain and obesity development. Additionally, obesity has also been associated with low bone quality^43^,^44^, which in turn has been associated with enhanced cartilage damage in OA animal models^45^.

Accordingly, to investigate the potential relationship between these pathologies, we focused on the study of the osteochondral junction because this interface plays a major role in OA development^8^,^10^,^46^. Abnormal mechanical loading on OA joints has been related to aberrant metabolism and turnover of the subchondral bone^8^,^10^. As a result, OA subchondral bone exhibits a complex structure with sclerotic and osteoporotic regions^45^,^46^. This heterogeneous structure affects the mechanical stress distribution on the cartilage, thereby promoting its degradation^46^. Accordingly, because cartilage and subchondral bone are thought to act as a functional unit^6^, and considering that the obesity-related adipokine LCN2, an articular catabolic adipokine, is highly induced in osteoblasts under an unloading stimulus^17^,^24^, we decided to investigate the role of LCN2 in osteoblast and chondrocyte metabolism by mimicking the OA osteochondral junction environment.

In this work we found that LCN2 expression was down-regulated throughout osteoblast differentiation. In contrast, LCN2 expression has been reported to increase during primary murine osteoblast differentiation^29^. However, in this work, Dx was added to the differentiation medium, which may explain the induction of LCN2. In fact, osteoblast de-differentiation promoted by a high Dx concentration^33^ induced LCN2 expression. Although this result suggests that LCN2 inhibition may be involved in osteoblast differentiation, none of our approaches involving treating or overexpressing LCN2 in MC3T3 cells induced any change in the expression of osteoblast differentiation markers (data not shown). Nonetheless, Rucci *et al*. have shown that LCN2 overexpression in human osteoblasts inhibits the expression of several differentiation markers^17^.

OA subchondral bone osteoblasts are exposed to multiple inflammatory factors, which may originate from other inflamed tissues or from osteoblasts exposed to an excessive mechanical loading or inflammatory stimuli^10^,^47^. According to this scenario, LCN2 expression is induced in murine and human osteoblasts upon stimulation with IL-1ß and a TLR4 agonist (LPS), thus suggesting that inflammation may induce LCN2 expression in OA subchondral bone osteoblasts. Interestingly, as found in chondrocytes^21^, despite the anti-inflammatory properties of Dx, osteoblast stimulation with Dx enhanced inflammation-mediated LCN2 expression, which has been recently associated with the cooperative recruitment of NFκB, C/EBPβ and the glucocorticoid receptor to the regulatory regions of LCN2 in an IκBζ-dependent manner^48^. Together, these results suggest that the use of corticoids in OA joints might contribute to the alteration of LCN2 metabolism in the subchondral bone.

Because inflammation strongly induces LCN2 expression in osteoblasts, and obesity and ageing, two major OA risk factors, are characterized by elevated LCN2 circulating levels, we investigated the effects of LCN2 on osteoblast metabolism. Although LCN2 has been shown to modulate cell viability in other cell types, including chondrocytes^23^,^26^,^27^, LCN2-enriched medium did not affect MC3T3 cell viability. Nonetheless, LCN2 has been shown to play a role in iron metabolism^34^, which has a strong effect on osteoblast viability^35^,^36^, and LCN2-enriched medium significantly enhanced the reduction of osteoblasts viability caused by iron overload. Supporting this role, LCN2 exhibits a similar mechanism in cell viability regulation in cardiomyocytes^49^.

Altogether, the effect of LCN2 on iron metabolism in osteoblasts and the ability of LNC2 to increase the activity of the bone resorption protease MMP-9^37^,^38^ strongly suggest that LCN2 is a new catabolic factor in bone tissue.

TGF-β1, a growth factor associated with bone growth^8^, is overexpressed in OA sclerotic subchondral bone, and its expression and activity are enhanced by mechanical stimulation^8^,^40^,^42^. Likewise, IGF-1 signalling is over-activated in OA sclerotic subchondral bone^50^, and its activity is blocked in bone exposed to an unloading stimulus^41^. Hence, different regions of the OA subchondral bone are associated with different activities of these growth factors. According to the catabolic activities of LCN2 described in this work, the observation that IGF-1 and TGF-β1 inhibited the inflammation-mediated LCN2 expression in human osteoblasts suggests that LCN2 inhibition might contribute to growth factor-mediated bone formation, which in turn may participate in the generation of OA subchondral bone sclerosis. Consistently with this idea, we also found that TGF-β1 inhibited LCN2 basal expression. Nonetheless, the confirmation of involvement of LCN2 in these alterations will require specific *in vivo* experiments showing an altered distribution of this adipokine in OA subchondral bone.

IGF-1 and TGF-β1 also play key roles in cartilage physiology and pathophysiology^8^,^51^. TGF-β1 participates in cartilage homeostasis^52^ and mediates the response of chondrocytes to mechanical loading^8^. Additionally, IGF-1 promotes chondrocyte proliferation and proteoglycan synthesis^53^,^54^. Given the catabolic activities of LCN2 on cartilage^5^,^22^, the observation that TGF-β1 inhibited the inflammation-mediated LCN2 expression in chondrocytes therefore suggests that TGF-β1 activity in cartilage might prevent the catabolic activities of LCN2. Likewise, IGF-1 may also prevent these catabolic activities because this growth factor also inhibited IL-1ß-mediated LCN2 expression in these cells. Nonetheless, it is notable that the OA environment (chondrocyte differentiation, ageing and oxidative stress) may disrupt the inhibitory properties of these growth factors^8^,^51^.

Biochemical and mechanical alterations in OA cartilage affect the OA subchondral bone and the ones in the OA subchondral bone affect the OA cartilage^6^. However, only osteoblasts induced the paracrine expression of LCN2 in chondrocytes, which suggests that the OA subchondral bone might contribute to increased LCN2 expression in the OA cartilage, thus linking the subchondral bone inflammatory state to cartilage catabolism.

**Figure 6:**
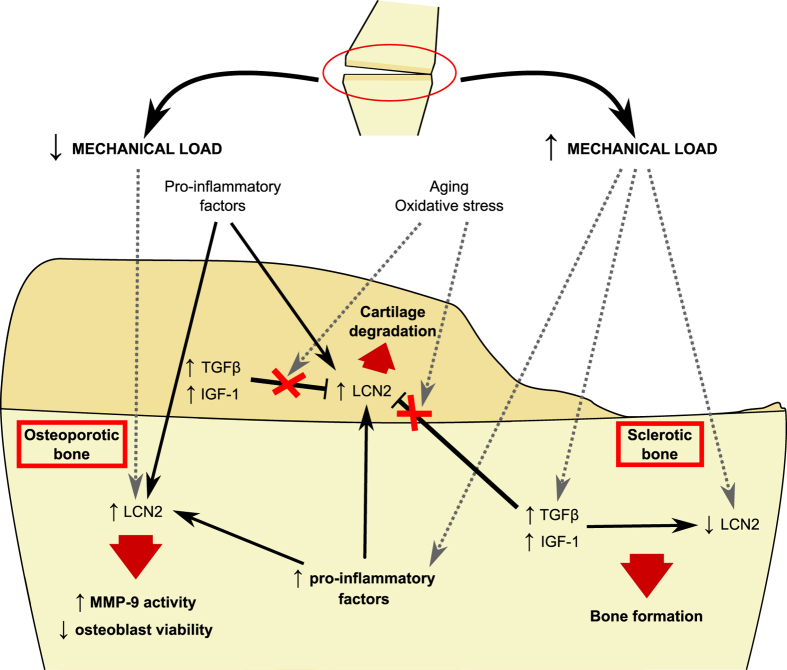
Speculative model of LCN2 regulation in the OA osteochondral junction. In the presence of alterations in articular biomechanics, joint tissues are subjected to different amounts of loading. Where the mechanical load is increased, LCN2 expression is decreased in bone, along with the up-regulation of growth factors, such as TGF-β1 and IGF-1, which might contribute to reduced LCN2 expression in osteoblasts. In this way, the bone exhibits higher bone formation and subchondral sclerosis. In areas of lower mechanical load, LCN2 expression is increased. In osteoblasts, this expression is enhanced by pro-inflammatory factors, which may originate from loaded bone and the inflamed environment. High LCN2 expression may have deleterious effects on bone, promoting MMP-9 activity and contributing to reduced osteoblast viability. Pro-inflammatory factors and other mediators derived from different joint tissues, including subchondral bone, may also induce LCN2 expression in the articular cartilage. Although TGF-β1 and IGF-1 also inhibit the induced expression of LCN2 in chondrocytes, impairment of their signalling pathways by oxidative stress, ageing, etc. may block this inhibition, thus leading to greater LCN2 expression and cartilage degradation during OA. Black lines indicate data described in this work, and grey dotted lines indicate data previously described^8^,^17^,^24^,^51^.

Finally, we propose a speculative model of LCN2 regulation in the OA osteochondral junction to help integrate the current knowledge of LCN2 modulation by mechanical stimuli in the bone, the data obtained in this work, and the diverse phenotypes of OA subchondral bone (Fig. 6). We suggest that the expression of LCN2 and its associated catabolic activities in the OA osteochondral junction might have a heterogeneous distribution. Hence, the maximum expression of this catabolic adipokine would be localized at regions exposed to the lowest mechanical loading and elevated inflammatory factors, and in environments with little or no growth factor signalling. We also suggest that the elevated circulating levels of LCN2 in obese and aged individuals might contribute to OA joint alterations^5^,^17^,^18^,^19^.

In conclusion, the data presented herein reveal a new facet of LCN2, the obesity-associated adipokine, as a catabolic factor in bone that is regulated in osteoblasts by inflammatory, catabolic, and anabolic factors. Additionally, we show that LCN2 expression in chondrocytes is regulated by osteoblasts through a paracrine mechanism and by the same factors that regulate its expression in osteoblasts. Overall, these results, together with the catabolic properties of LCN2 in other joint tissues, suggest that LCN2 is an active catabolic agent in OA joints that serves as a link among obesity, ageing and OA joint alterations.

## Additional Information

**How to cite this article**: Villalvilla, A. *et al*. The adipokine lipocalin-2 in the context of the osteoarthritic osteochondral junction. *Sci. Rep*. **6**, 29243; doi: 10.1038/srep29243 (2016).

## Supplementary Material

Supplementary Information

## References

[b1] Lee J. . Sedentary behavior and physical function: objective evidence from the Osteoarthritis Initiative. Arthritis Care Res 67, 366–373 (2015). 10.1002/acr.22432 PMC4336845 25155652

[b2] Shields M. & Tremblay M. S. Sedentary behaviour and obesity. Health Rep 19, 19–30 (2008). 18642516

[b3] Findlay D. M. & Atkins G. J. Osteoblast-chondrocyte interactions in osteoarthritis. Curr Osteoporos Rep 12, 127–134 (2014). 24458429 10.1007/s11914-014-0192-5 PMC3933767

[b4] Creamer P. & Hochberg M. C. Osteoarthritis. Lancet 350, 503–508 (1997). 9274595 10.1016/S0140-6736(97)07226-7

[b5] Gómez R. . What’s new in our understanding of the role of adipokines in rheumatic diseases? Nat Rev Rheumatol 7, 528–536 (2011). 21808287 10.1038/nrrheum.2011.107

[b6] Lories R. J. & Luyten F. P. The bone-cartilage unit in osteoarthritis. Nat Rev Rheumatol 7, 43–49 (2011). 21135881 10.1038/nrrheum.2010.197

[b7] Villalvilla A., Gómez R., Largo R. & Herrero-Beaumont G. Lipid transport and metabolism in healthy and osteoarthritic cartilage. Int J Mol Sci 14, 20793–20808 (2013). 24135873 10.3390/ijms141020793 PMC3821643

[b8] Zhen G. & Cao X. Targeting TGFβ signaling in subchondral bone and articular cartilage homeostasis. Trends Pharmacol Sci 35, 227–236 (2014). 24745631 10.1016/j.tips.2014.03.005 PMC4058854

[b9] Wu D. D., Burr D. B., Boyd R. D. & Radin E. L. Bone and cartilage changes following experimental varus or valgus tibial angulation. J Orthop Res 8, 572–585 (1990). 2355297 10.1002/jor.1100080414

[b10] Sanchez C. . Regulation of subchondral bone osteoblast metabolism by cyclic compression. Arthritis Rheum 64, 1193–1203 (2012). 22034083 10.1002/art.33445

[b11] Gómez R., Villalvilla A., Largo R., Gualillo O. & Herrero-Beaumont G. TLR4 signalling in osteoarthritis-finding targets for candidate DMOADs. Nat Rev Rheumatol 11, 159–170 (2015). 25512010 10.1038/nrrheum.2014.209

[b12] Sharma A. R., J. S., L. S.-S. & N. J.-S. Interplay between Cartilage and Subchondral Bone Contributing to Pathogenesis of Osteoarthritis. Int J Mol Sci 14, 19805–19830 (2013). 24084727 10.3390/ijms141019805 PMC3821588

[b13] Daheshia M. & Yao J. Q. The interleukin 1beta pathway in the pathogenesis of osteoarthritis. J Rheumatol 35, 2306–2312 (2008). 18925684 10.3899/jrheum.080346

[b14] Sanchez C. . Osteoblasts from the sclerotic subchondral bone downregulate aggrecan but upregulate metalloproteinases expression by chondrocytes. This effect is mimicked by interleukin-6, -1beta and oncostatin M pre-treated non-sclerotic osteoblasts. Osteoarthr Cartil 13, 979–987 (2005). 16243232 10.1016/j.joca.2005.03.008

[b15] van der Kraan P. M. & van den Berg W. B. Chondrocyte hypertrophy and osteoarthritis: role in initiation and progression of cartilage degeneration? Osteoarthr Cartil 20, 223–232 (2012). 22178514 10.1016/j.joca.2011.12.003

[b16] Dvinge H. & Bertone P. A meta-analysis of sex differences prevalence, incidence and severity of osteoarthritis. Osteoarthr Cartil 13, 769–781 (2005). 15978850 10.1016/j.joca.2005.04.014

[b17] Rucci N. . Lipocalin 2: a new mechanoresponding gene regulating bone homeostasis. J Bone Miner Res 30, 357–368 (2015). 25112732 10.1002/jbmr.2341

[b18] Lim W. H. . Circulating Lipocalin 2 Levels Predict Fracture-Related Hospitalizations in Elderly Women: A Prospective Cohort Study. J Bone Miner Res 30, 2078–2085 (2015). 25939604 10.1002/jbmr.2546

[b19] Panidis D. . The effects of obesity and polycystic ovary syndrome on serum lipocalin-2 levels: a cross-sectional study. Reproductive Biology and Endocrinology 8, 151 (2010). 21143924 10.1186/1477-7827-8-151 PMC3004902

[b20] Triebel S., Bläser J., Reinke H. & Tschesche H. A 25 kDa alpha 2-microglobulin-related protein is a component of the 125 kDa form of human gelatinase. FEBS Lett 314, 386–388 (1992). 1281792 10.1016/0014-5793(92)81511-j

[b21] Conde J. . Expanding the adipokine network in cartilage: identification and regulation of novel factors in human and murine chondrocytes. Ann Rheum Dis 70, 551–559 (2011). 21216818 10.1136/ard.2010.132399

[b22] Gupta K., Shukla M., Cowland J. B., Malemud C. J. & Haqqi T. M. Neutrophil gelatinase–associated lipocalin is expressed in osteoarthritis and forms a complex with matrix metalloproteinase 9. Arthritis Rheum 56, 3326–3335 (2007). 17907186 10.1002/art.22879

[b23] Katano M. . Implication of granulocyte-macrophage colony-stimulating factor induced neutrophil gelatinase-associated lipocalin in pathogenesis of rheumatoid arthritis revealed by proteome analysis. Arthritis Res Ther 11, R3 (2009). 20527084 10.1186/ar2587 PMC2688233

[b24] Capulli M., Rufo A., Teti A. & Rucci N. Global transcriptome analysis in mouse calvarial osteoblasts highlights sets of genes regulated by modeled microgravity and identifies a ‘mechanoresponsive osteoblast gene signature’. J Cell Biochem 107, 240–252 (2009). 19288527 10.1002/jcb.22120

[b25] Zerega B., Cermelli S., Michelis B., Cancedda R. & Cancedda F. D. Expression of NRL/NGAL (neu-related lipocalin/neutrophil gelatinase-associated lipocalin) during mammalian embryonic development and in inflammation. Eur J Cell Biol 79, 165–172 (2000). 10777108 10.1078/s0171-9335(04)70019-9

[b26] Owen H. C., Roberts S. J., Ahmed S. F. & Farquharson C. Dexamethasone-induced expression of the glucocorticoid response gene lipocalin 2 in chondrocytes. Am J Physiol Endocrinol Metab 294, E1023–E1034 (2008). 18381927 10.1152/ajpendo.00586.2007

[b27] Gómez R. . Nitric oxide boosts TLR-4 mediated lipocalin 2 expression in chondrocytes. J Orthop Res 31, 1046–1052 (2013). 23483583 10.1002/jor.22331

[b28] Shen F., Ruddy M. J., Plamondon P. & Gaffen S. L. Cytokines link osteoblasts and inflammation: microarray analysis of interleukin-17- and TNF-alpha-induced genes in bone cells. J Leukoc Biol 77, 388–399 (2005). 15591425 10.1189/jlb.0904490

[b29] Costa D. . Altered bone development and turnover in transgenic mice over-expressing lipocalin-2 in bone. J Cell Physiol 228, 2210–2221 (2013). 23606520 10.1002/jcp.24391

[b30] Villalvilla A. . 6-Shogaol inhibits chondrocytes’ innate immune responses and cathepsin-K activity. Mol Nutr Food Res 58, 256–266 (2014). 24039109 10.1002/mnfr.201200833

[b31] Zhen G. . Inhibition of TGF-β signaling in mesenchymal stem cells of subchondral bone attenuates osteoarthritis. Nat Med 19, 704–712 (2013). 23685840 10.1038/nm.3143 PMC3676689

[b32] Yan Q.-W. . The adipokine lipocalin 2 is regulated by obesity and promotes insulin resistance. Diabetes 56, 2533–2540 (2007). 17639021 10.2337/db07-0007

[b33] Luppen C. A., Smith E., Spevak L., Boskey A. L. & Frenkel B. Bone morphogenetic protein-2 restores mineralization in glucocorticoid-inhibited MC3T3-E1 osteoblast cultures. J Bone Miner Res 18, 1186–1197 (2003). 12854828 10.1359/jbmr.2003.18.7.1186

[b34] Yang J. . An iron delivery pathway mediated by a lipocalin. Mol Cell 10, 1045–1056 (2002). 12453413 10.1016/s1097-2765(02)00710-4

[b35] Yamasaki K. & Hagiwara H. Excess iron inhibits osteoblast metabolism. Toxicology Letters 191, 211–215 (2009). 19735707 10.1016/j.toxlet.2009.08.023

[b36] Zhao G.-Y. . A Comparison of the Biological Activities of Human Osteoblast hFOB1.19 Between Iron Excess and Iron Deficiency. Biol Trace Elem Res 150, 487–495 (2012). 23054865 10.1007/s12011-012-9511-9

[b37] Vu T. H. . MMP-9/gelatinase B is a key regulator of growth plate angiogenesis and apoptosis of hypertrophic chondrocytes. Cell 93, 411–422 (1998). 9590175 10.1016/s0092-8674(00)81169-1 PMC2839071

[b38] Nyman J. S. . Differential effects between the loss of MMP-2 and MMP-9 on structural and tissue-level properties of bone. J Bone Miner Res 26, 1252–1260 (2011). 21611966 10.1002/jbmr.326 PMC3312757

[b39] Monroe D. G., Mcgee-Lawrence M. E., Oursler M. J. & Westendorf J. J. Update on Wnt signaling in bone cell biology and bone disease. Gene 492, 1–18 (2012). 22079544 10.1016/j.gene.2011.10.044 PMC3392173

[b40] Klein-Nulend J., Roelofsen J., Sterck J. G., Semeins C. M. & Burger E. H. Mechanical loading stimulates the release of transforming growth factor-beta activity by cultured mouse calvariae and periosteal cells. J Cell Physiol 163, 115–119 (1995). 7896887 10.1002/jcp.1041630113

[b41] Bikle D. D., Halloran B. P. & Morey-Holton E. Impact of skeletal unloading on bone formation: role of systemic and local factors. Acta Astronaut 33, 119–129 (1994). 11539511 10.1016/0094-5765(94)90116-3

[b42] Matziolis D. . Osteogenic predifferentiation of human bone marrow-derived stem cells by short-term mechanical stimulation. Open Orthop J 5, 1–6 (2011). 21270950 10.2174/1874325001105010001 PMC3027083

[b43] Pollock N. K. . Is adiposity advantageous for bone strength? A peripheral quantitative computed tomography study in late adolescent females. Am. J. Clin. Nutr. 86, 1530–1538 (2007). 17991669 10.1093/ajcn/86.5.1530

[b44] Zhao L.-J. . Correlation of obesity and osteoporosis: effect of fat mass on the determination of osteoporosis. J Bone Miner Res 23, 17–29 (2008). 17784844 10.1359/JBMR.070813 PMC2663586

[b45] Herrero-Beaumont G. & Roman-Blas J. A. Osteoarthritis: Osteoporotic OA: a reasonable target for bone-acting agents. Nat Rev Rheumatol 9, 448–450 (2013). 23857129 10.1038/nrrheum.2013.113

[b46] Chiba K. . Osteoporotic changes of subchondral trabecular bone in osteoarthritis of the knee: a 3-T MRI study. Osteoporos Int 23, 589–597 (2012). 21359670 10.1007/s00198-011-1585-2

[b47] Baum R. & Gravallese E. M. Impact of inflammation on the osteoblast in rheumatic diseases. Curr Osteoporos Rep 12, 9–16 (2014). 24363057 10.1007/s11914-013-0183-y PMC3943531

[b48] Yamazaki S., Akira S. & Sumimoto H. Glucocorticoid augments lipopolysaccharide-induced activation of the IκBζ-dependent genes encoding the anti-microbial glycoproteins lipocalin 2 and pentraxin 3. J Biochem 157, 399–410 (2015). 25552549 10.1093/jb/mvu086

[b49] Xu G. . Lipocalin-2 induces cardiomyocyte apoptosis by increasing intracellular iron accumulation. J Biol Chem 287, 4808–4817 (2012). 22117066 10.1074/jbc.M111.275719 PMC3281654

[b50] Massicotte F. . Abnormal insulin-like growth factor 1 signaling in human osteoarthritic subchondral bone osteoblasts. Arthritis Res Ther 8, R177 (2006). 17129375 10.1186/ar2087 PMC1794522

[b51] Loeser R. F., Gandhi U., Long D. L., Yin W. & Chubinskaya S. Aging and oxidative stress reduce the response of human articular chondrocytes to insulin-like growth factor 1 and osteogenic protein 1. Arthritis Rheumatol 66, 2201–2209 (2014). 24664641 10.1002/art.38641 PMC4116467

[b52] Shen J. . Deletion of the transforming growth factor β receptor type II gene in articular chondrocytes leads to a progressive osteoarthritis-like phenotype in mice. Arthritis Rheum 65, 3107–3119 (2013). 23982761 10.1002/art.38122 PMC3928444

[b53] Guerne P. A., Blanco F., Kaelin A., Desgeorges A. & Lotz M. Growth factor responsiveness of human articular chondrocytes in aging and development. Arthritis Rheum 38, 960–968 (1995). 7612045 10.1002/art.1780380712

[b54] DeGroot J. . Age-related decrease in proteoglycan synthesis of human articular chondrocytes: the role of nonenzymatic glycation. Arthritis Rheum 42, 1003–1009 (1999). 10323457 10.1002/1529-0131(199905)42:5<1003::AID-ANR20>3.0.CO;2-K

