# Corrigendum: The adipokine lipocalin-2 in the context of the osteoarthritic osteochondral junction

**DOI:** 10.1038/srep30666

**Published:** 2016-08-19

**Authors:** Amanda Villalvilla, Adela García-Martín, Raquel Largo, Oreste Gualillo, Gabriel Herrero-Beaumont, Rodolfo Gómez

Scientific Reports 6: Article number: 2924310.1038/srep29243; published online: 07 07 2016; updated: 08 19 2016.

The Acknowledgements section in this Article is incomplete.

“The authors acknowledge Mr. Oliver Shaw for performing the English revision and the support of Dr. Esbrit’s. The authors’ research is supported by research grants from Fondo de Investigación Sanitaria funded by the Instituto de Salud Carlos III (PI12/00144, PI13/00570, CP15/00007, PI14/00016 and PIE13/00024). R.G. is funded by the Instituto de Salud Carlos III through a Miguel Servet programme. A.V. is the recipient of a fellowship from the Fundación Conchita Rábago. A.G.M. was funded by the Universidad Carlos III de Madrid (Spain). R.L. and O.G. were funded by the Instituto de Salud Carlos III. O.G. is a member of the RETICS Programme, RD12/0009/0008 Instituto de Salud Carlos III (ISCIII).”

should read:

“The authors acknowledge Mr. Oliver Shaw for performing the English revision and the support of Dr. Esbrit’s. The authors’ research is supported by research grants from Fondo de Investigación Sanitaria funded by the Instituto de Salud Carlos III (PI12/00144, PI13/00570, CP15/00007, PI14/00016 and PIE13/00024). R.G. is funded by the Instituto de Salud Carlos III through a Miguel Servet programme. A.V. is the recipient of a fellowship from the Fundación Conchita Rábago. A.G.M. was funded by the Universidad Carlos III de Madrid (Spain). R.L. and O.G. were funded by the Instituto de Salud Carlos III. O.G. is a member of the RETICS Programme, RD12/0009/0008 Instituto de Salud Carlos III (ISCIII). The research is supported by research grant from FEDER.”

