# Supplementary material for: The adipokine lipocalin-2 in the context of the osteoarthritic osteochondral junction
Source: Sci Rep. 2016 Jul 7;6:29243. doi: 10.1038/srep29243 (PMC4935838; doi:10.1038/srep29243)
Supplement: Supplementary Information [file srep29243-s1.pdf]

The adipokine lipocalin-2 in the context of the osteoarthritic osteochondral junction

Amanda Villalvilla[1], Adela García-Martín[3], Raquel Largo[1], Oreste Gualillo[4], Gabriel Herrero-Beaumont*[1] & Rodolfo Gómez[2]

[1] Bone and Joint Research Unit, IIS-Fundación Jiménez Díaz, UAM, Avda Reyes Católicos, Madrid, 28040, Spain.

[2] Musculoskeletal Pathology Laboratory, Institute IDIS, Santiago University Clinical Hospital, Santiago de Compostela, 15706, Spain.

[3] Department of Bioengineering, Universidad Carlos III de Madrid. CIEMAT-CIBERER. IIS-Fundación Jiménez Díaz, Madrid, 28040, Spain.

[4] Research Laboratory 9 (NEIRID LAB), Institute of Medical Research, SERGAS, Santiago University Clinical Hospital, Santiago de Compostela, 15706, Spain.

A

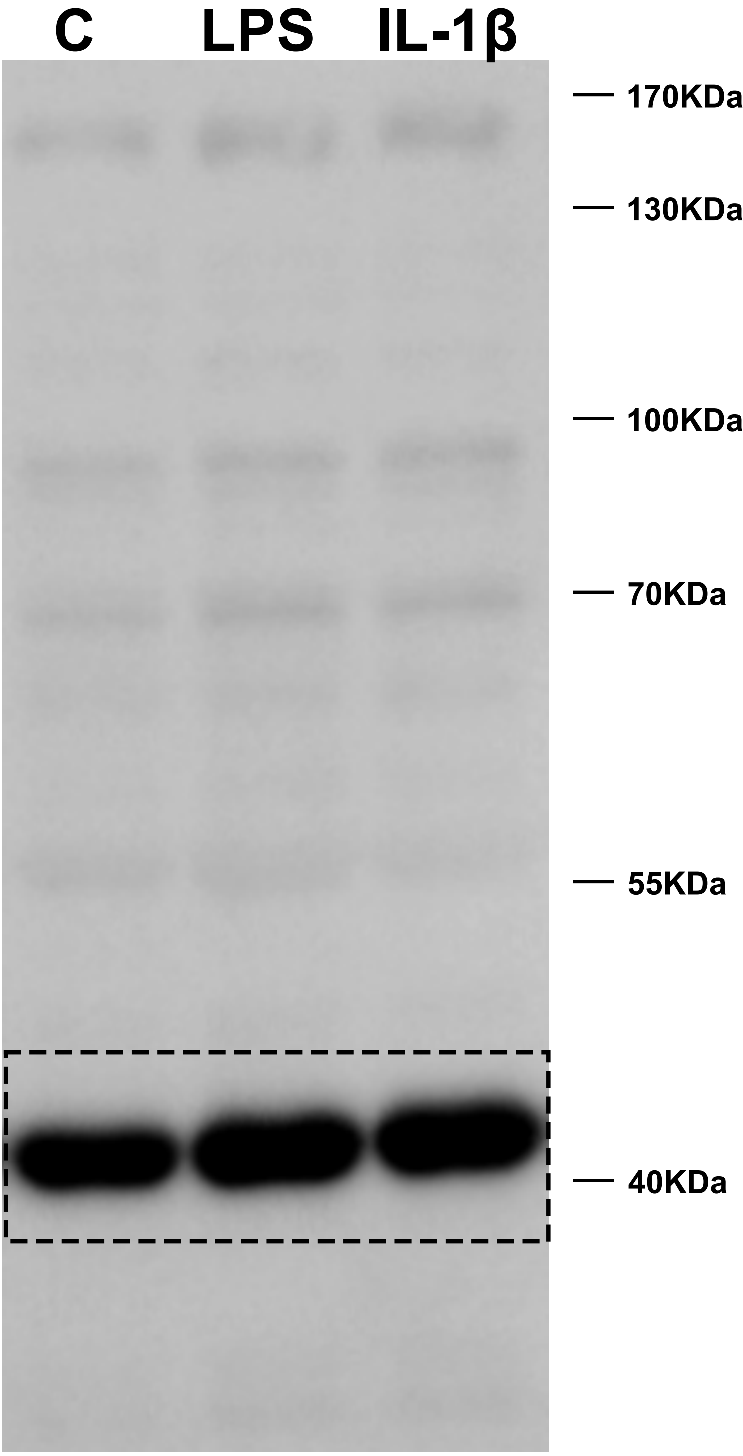

C
LPS
IL-1β
170KDa
130KDa
100KDa
70KDa
55KDa
40KDa

B

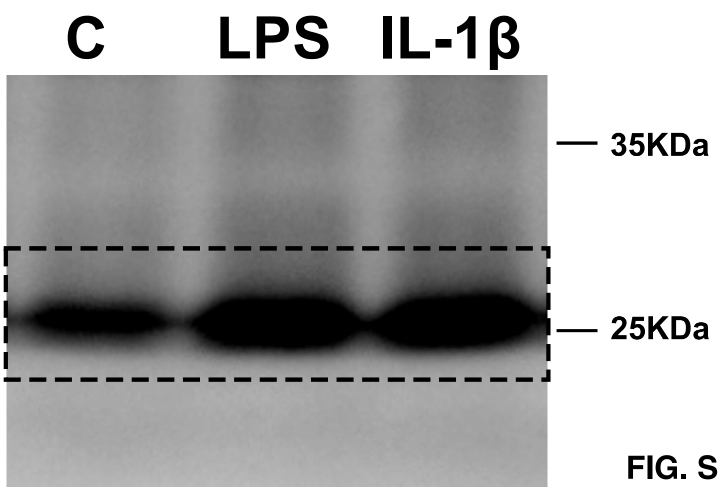

C
LPS
IL-1β
35KDa
25KDa

FIG. S1

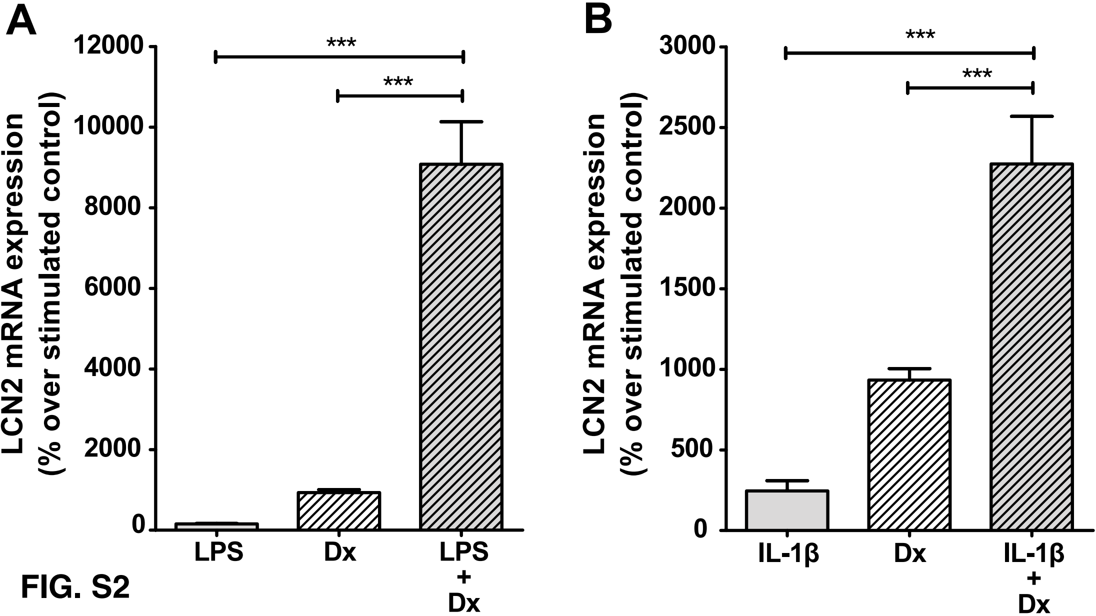
A
LCN2 mRNA expression
(% over stimulated control)
12000
10000
8000
6000
4000
2000
0
***
***
LPS
Dx
LPS
+
Dx
B
LCN2 mRNA expression
(% over stimulated control)
3000
2500
2000
1500
1000
500
0
***
***
IL-1β
Dx
IL-1β
+
Dx

FIG. S2

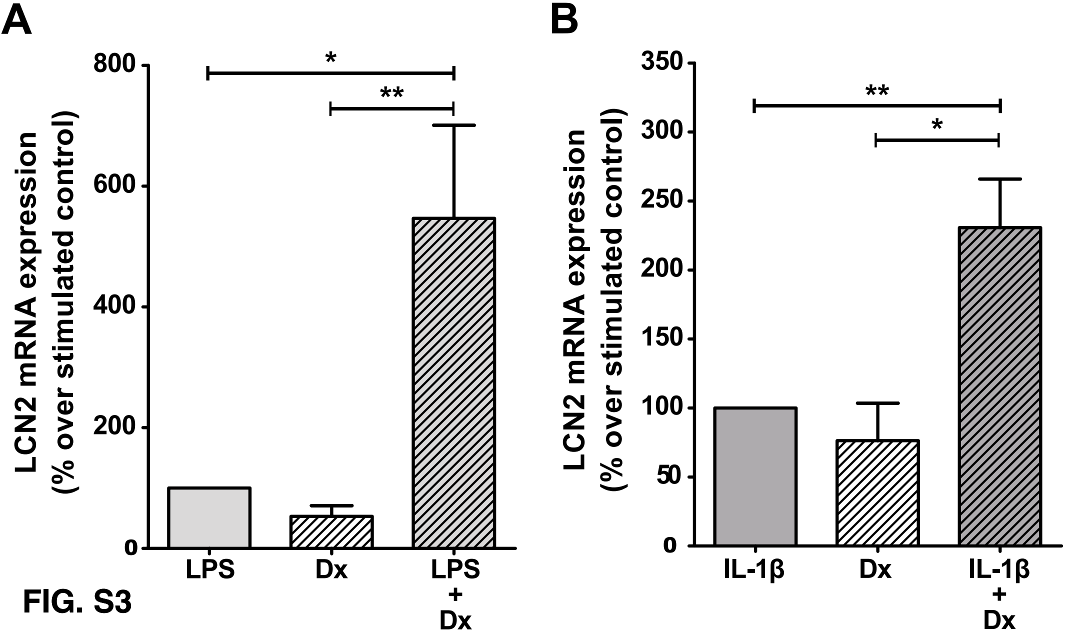
A
B
LCN2 mRNA expression
(% over stimulated control)
800
600
400
200
0
*
**
LPS
Dx
LPS
+
Dx
350
300
250
200
150
100
50
0
**
*
IL-1β
Dx
IL-1β
+
Dx

FIG. S3

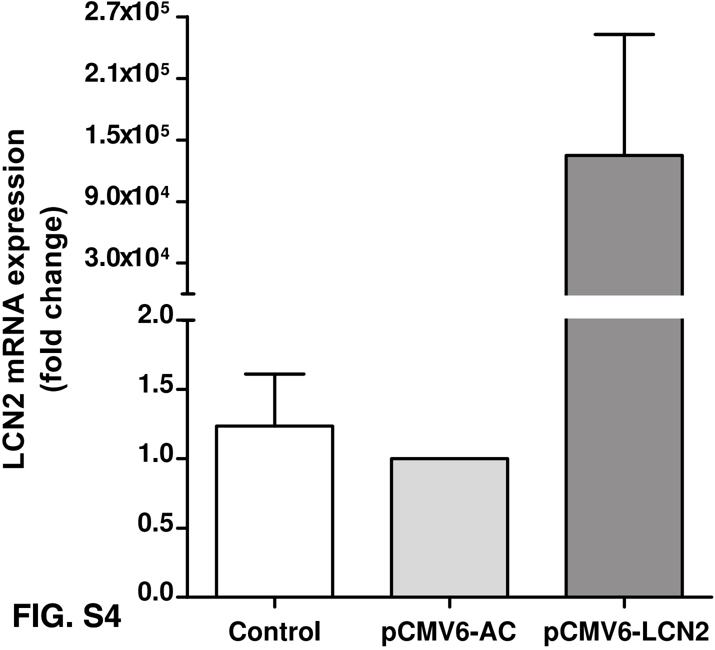

2.7x10^5
2.1x10^5
1.5x10^5
9.0x10^4
3.0x10^4
2.0
1.5
1.0
0.5
0.0
LCN2 mRNA expression (fold change)
Control
pCMV6-AC
pCMV6-LCN2

FIG. S4

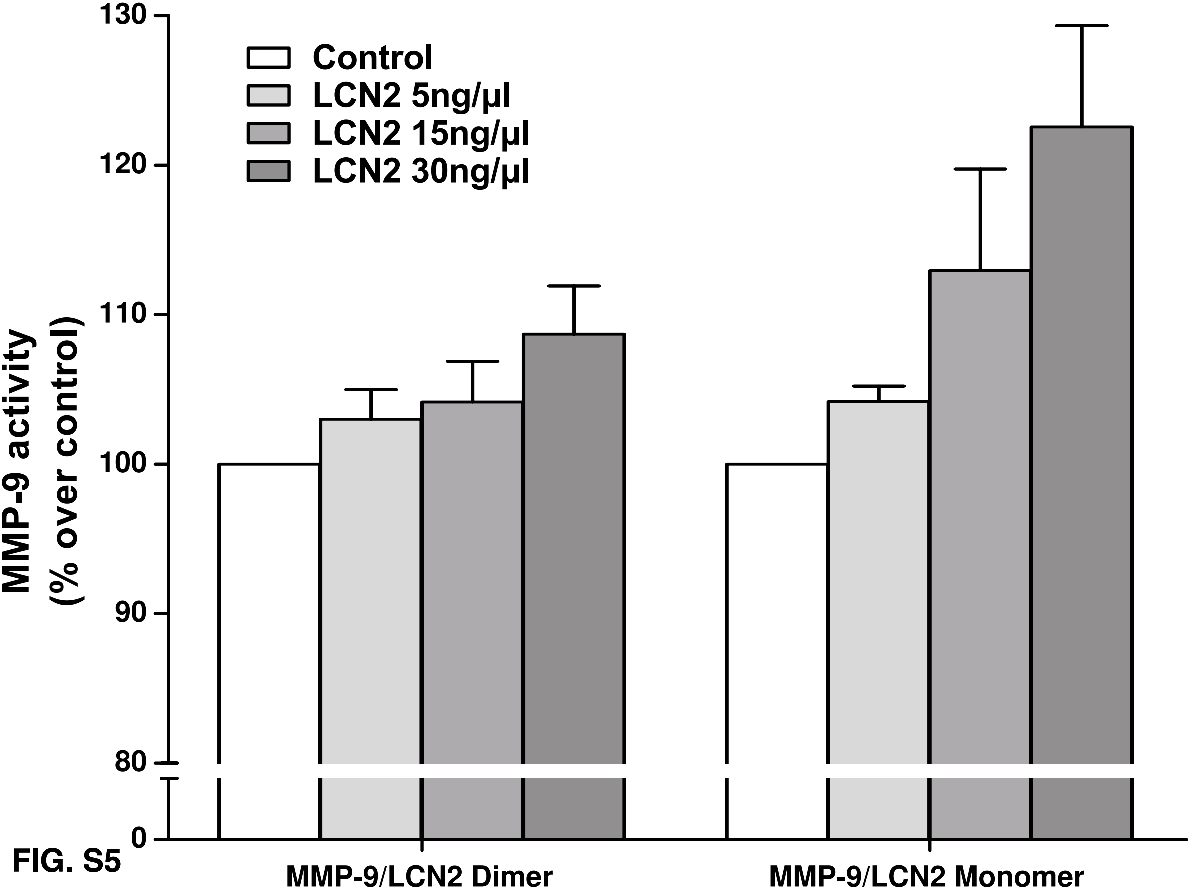
Control
LCN2 5ng/µl
LCN2 15ng/µl
LCN2 30ng/µl
MMP-9 activity
(% over control)
130
120
110
100
90
80
0
MMP-9/LCN2 Dimer
MMP-9/LCN2 Monomer

FIG. S5

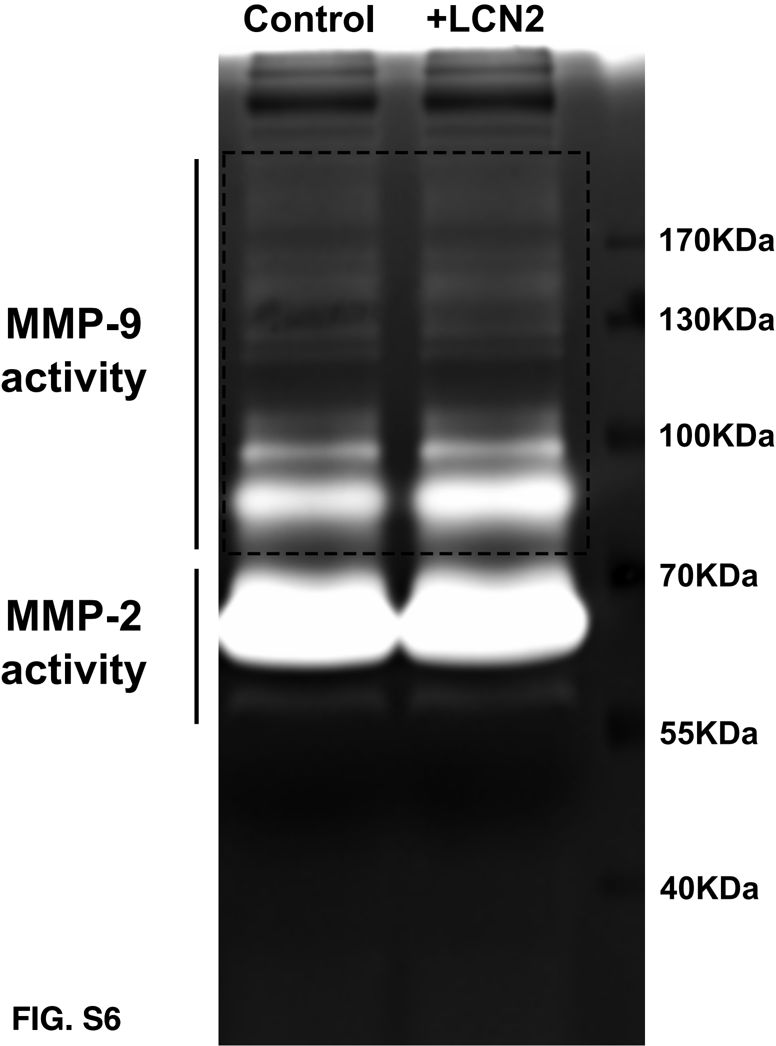
Control
+LCN2
MMP-9 activity
MMP-2 activity
170KDa
130KDa
100KDa
70KDa
55KDa
40KDa

FIG. S6

Supplementary Fig. 1. Induction of LCN2 protein expression in human osteoblasts.

Full-length blots of the cropped blots for actin and LCN2 presented in Figure 2C. The cropped region is delimited by a dotted line. All the gels were run under the same experimental conditions.

Supplementary Fig. 2. Modulation of LCN2 gene expression in MC3T3 cells by pro-inflammatory and catabolic factors. After overnight culture in serum-free conditions, non-differentiated MC3T3 cells were stimulated with 1 μg/ml LPS (A) or 1 ng/ml IL-1β (B) in combination with 10 μM Dx for 48 h, and LCN2 mRNA expression was measured by real-time PCR. The results are presented as the mean ± SEM of at least three independent experiments and expressed as the percentage of the stimulated control. ***p<0.001.

Supplementary Fig. 3. Modulation of LCN2 gene expression in primary human osteoblasts by pro-inflammatory and catabolic factors. After overnight culture in 1% FBS, primary human osteoblasts were stimulated with 10 μM Dx alone or in combination with 1 μg/ml LPS (A) or 1 ng/ml IL-1β (B) for 48 h, and LCN2 gene expression was measured by real-time PCR. The results are presented as the mean ± SEM of at least four independent experiments and expressed as the percentage of the stimulated control. *p<0.05; **p<0.01; ***p<0.001

Supplementary Fig. 4. Overexpression of LCN2 in MC3T3 cells. MC3T3 cells were transfected with pCMV6-LCN2 or the empty vector (pCMV6-AC), and LCN2 gene

expression was evaluated by real-time PCR at 48 h post-transfection. The results are presented as the mean ± SEM of at least three independent experiments.

Supplementary Fig. 5. Stabilization of MMP-9 activity by LCN2. CM from MC3T3 cells was incubated with 5, 15 or 30 ng/µl LCN2 for 1 h at 37ºC. Then, the gelatinolytic activity of the MMP-9/LCN2 complex (monomer and dimer) was evaluated by gelatine zymography. The results are presented as the mean ± SEM of at least three independent experiments.

Supplementary Fig. 6. Effect of LCN2 on MMP-9 activity.

A full-length gel of the cropped gelatine zymography presented in Figure 3C. MMP-9 and MMP-2 activity are shown. The cropped region is delimited by a dotted line. All the gels were run under the same experimental conditions.
